# Corrosion Behavior of SiMo Cast Iron under Controlled Conditions

**DOI:** 10.3390/ma15093225

**Published:** 2022-04-29

**Authors:** Marcin Stawarz, Paweł M. Nuckowski

**Affiliations:** 1 Department of Foundry Engineering, Faculty of Mechanical Engineering, Silesian University of Technology, 7 Towarowa Street, 44-100 Gliwice, Poland; 2 Materials Research Laboratory, Faculty of Mechanical Engineering, Silesian University of Technology, 18A Konarskiego Street, 44-100 Gliwice, Poland; pawel.nuckowski@polsl.pl

**Keywords:** corrosion, SiMo cast iron, hematite, magnetite, iron carbide

## Abstract

The resistance of cast iron to chemical and electrochemical corrosion depends on the exposure conditions, chemical composition and the distribution of alloying elements in the microstructure. The article presents an attempt to describe the formation of a corrosion layer under controlled chemical corrosion conditions for SiMo ductile iron. In the experiment, a (HT-HRD) chamber for heating the samples with a controlled atmosphere was used, at the same time providing the possibility of testing the sample surface using the XRD method. The analysis was both qualitative and quantitative. It allowed us to capture the sequence of the oxide layer growth on the tested samples. The beneficial effect of molybdenum on the corrosion resistance of SiMo cast iron has been proven. For all cases under analysis, the phenomenon of an increase in the share of hematite (Fe_2_O_3_) and magnetite (Fe_3_O_4_) was observed in the subsequent sample heating cycles. It turned out that the addition of molybdenum helps to create a tight oxide coating that inhibits the further growth of corrosion processes. Increasing the share of molybdenum in the alloy also reduces the thickness of the oxide layer.

## 1. Introduction

Many components in the automotive industry are still manufactured as castings. One of the examples is an exhaust manifold made of SiMo cast iron. This material is also used to manufacture other components exposed to high temperatures (e.g., accessories in heat-treatment furnaces: hooks, slings, racks). Iron-based cast parts are readily used because they are relatively cheap and display good mechanical properties. Corrosion phenomena and elevated operating temperatures of such components are a certain problem here. The resistance of cast iron to chemical and electrochemical corrosion depends mainly on the chemical composition and the distribution of alloying elements in the microstructure. The authors Xiang, S., et al. [1], Çelik, G.A., et al. [2], Ebel, A., et al. [3], Lekakh, S.N., et al. [4], Tholence, F., et al. [5,6,7,8], Yang, Y.L., et al. [9] and Choe, K.H. with their team [10] believe that the type of metallic matrix and its chemical composition are important in the high-temperature oxidation behaviour of SiMo ferritic ductile cast iron. In the process of corrosion with oxygen depolarisation, the diffusion of molecular oxygen towards the cast iron surface plays an essential role. The corrosion rate is limited by the inflow of atmospheric oxygen to the corrosive medium [11]. The presence of numerous phases with different potentials in the alloy and the formation of microcells significantly increase the corrosion rate, especially when these phases differ significantly in terms of electrode potential. Such conditions exist in cast iron whose basic components of microstructure show significant electric potential differences. This can be seen in the voltage series defined [12] in relation to the calomel electrode in a 1% NaCl solution. The electrolytic iron has a potential of −0.755 V, while the potential of graphite is positive and is 0.372 V. This comparison shows that the difference in electric potential between iron and graphite is about 1 V, which favours the transfer of microstructure graphite adjacent components to the solution. Taking into account the role of graphite in the formation of microcells, it is believed that the spheroidal form of graphite is preferable due to the lower degree of surface development compared to flake graphite [13], with the same graphite share in both cases. For cast iron, the presence of microcells does not play a particularly important role in increasing the corrosion rate, because the graphite layer can protect the cast iron from corrosive processes. The corrosion resistance is also positively affected by the high tightness of castings [14,15,16], especially the lack of gas porosity and slag inclusion [17], work temperature [18,19], graphite shape participation [20], thermomechanical conditions [21,22,23] environmental aspects [24], bifilm inclusions [25,26,27,28,29] and decarburization process [4].

The diffusion of oxygen under the operating conditions of the SiMo cast iron exhaust manifold causes the pores left after the spherical graphite precipitates to be filled with oxide scale, hence the amount and morphology of graphite in the casting matrix is important. In addition, too much carbon in the alloy causes the unfavourable phenomenon of graphite flotation [30,31,32]. On the other hand, the iron and silicon in the SiMo composition tend to diffuse to the outside to form an outer oxide layer (Fe_2_O_3_, Fe_3_O_4_) and an inner oxide layer (Fe_2_SiO_4_), which provides a protective effect [4,7,10].

Considering the above mentioned, it is important to analyse the very mechanism of the oxide layer formation on the surface of the casting using the high-temperature X-ray diffraction technique (HT-XRD). The solution proposed in the article allows us to analyse the formation of a passive layer on the casting surface under strictly controlled conditions. At the same time, we can observe the growth of the oxide layer both in terms of its chemical composition as well as in quantitative approach.

## 2. Materials and Methods

The experimental melts were conducted in the induction furnace with a medium frequency and a capacity of 25 kg according to [17]. The charge consisted of scrap steel with a low sulphur content. Other ingredients added during the melting were ferrosilicon FeSi75, synthetic graphite of carbon content greater than 99.35% and a rich alloy in FeMo65. The cast iron spheroidization process was conducted in the bottom of the ladle, after the nodulizing agent was covered by pieces of steel scrap. The magnesium-rich alloy used in the studies was FeSiMg5RE [17].

In the studies, samples of spheroidal cast iron with Si content of 5% and Mo content of 0–2% were used. The chemical composition was determined on the basis of the Leco spectrometer (Model No 607–500, Leco Corporation, 3000 Lakeview Ave, St. Joseph, MI, USA) and the Leco carbon and sulphur analyser CS-125 (Leco Corporation, 3000 Lakeview Ave, St. Joseph, MI, USA). The chemical compositions of the tested samples are presented in Table 1.

The samples for metallographic tests were cast from the experimental melts. These samples were cast in resin-coated sand moulds, the shape of which corresponds to the standardized samples to determine the impact toughness. Metallographic tests were performed using scanning electron microscopy (Phenom Pro-X with EDS system—PhenomWorld B.V. Dillenburgstraat 9T, Eindhoven, The Netherlands).

The sections of the samples analysed were included in the conductive resin (Konductomet 1 Black, Agar Scientific Ltd., Stansted, UK).

**Figure 1:**
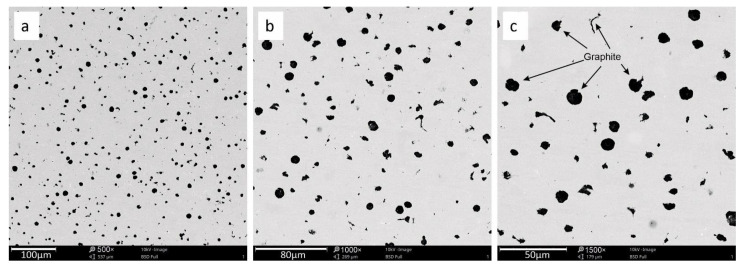
The cast iron microstructure for sample 1 with precipitation of nodular and vermicular graphite. Magnification 500× (**a**), 1000× (**b**), 1500× (**c**).

**Figure 2:**
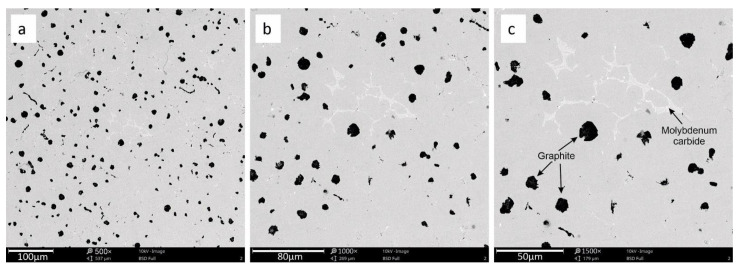
The cast iron microstructure for sample 2 with precipitation of graphite and molybdenum carbide. Magnification 500× (**a**), 1000× (**b**), 1500× (**c**).

**Figure 3:**
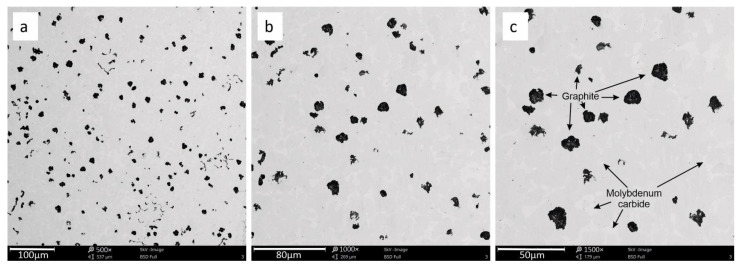
The cast iron microstructure for sample 3 with precipitation of graphite and molybdenum carbide. Magnification 500× (**a**), 1000× (**b**), 1500× (**c**).

The microstructures of the investigated cast iron are presented below. There are visible particles of spheroidal graphite and several particles of vermicular graphite as shown in Figure 1, Figure 2 and Figure 3 additionally show the precipitation of molybdenum carbide, which appeared in the metallic matrix in the form of bright particles located on the grain. The limit distribution of molybdenum carbide grains in SiMo cast iron is a known phenomenon, described in the literature by [6,7,8,9,10,11].

**Figure 4:**
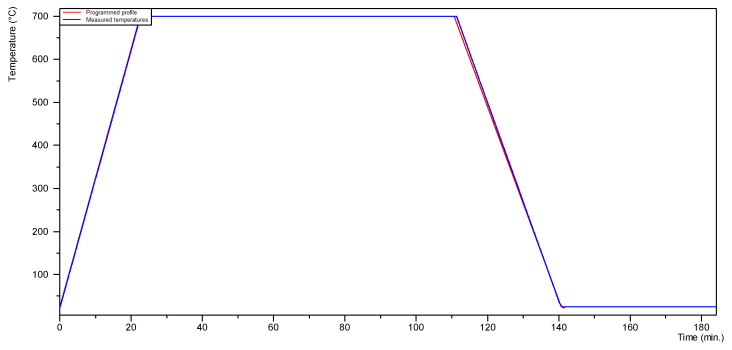
Parameters of the oxidation process in a single cycle: oxidation temperature 700 °C, heating rate 60 °C per min, holding time 90 min, cooling rate 23 °C per min.

In order to assess the mechanism of oxide layer formation on the surface of SiMo castings, the high-temperature X-ray diffraction (HT-XRD) technique was applied. The oxidation and X-ray diffraction (XRD) measurements were taken by using a Panalytical X’Pert Pro MPD diffractometer (Almelo, The Netherlands) equipped with a high-temperature chamber Anton Paar HTK 16 (Graz, Austria) with a platinum heating unit. The oxidation process was conducted at 700 °C, in cycles (from 1 to 3 cycles) for each SiMo sample, with free air flow to the HTK chamber. The parameters of the oxidation process in a single cycle are shown in Figure 4. After each cycle, diffraction tests were performed in the Bragg–Brentano geometry within the angular range from 10 to 120° 2θ, step 0.026° and time per step 100 s, using the filtered radiation of a cobalt X-ray lamp (Co_Kα_ λ = 0.179 nm). The X-ray qualitative phase analysis was performed using the dedicated Panalytical High Score Plus (v. 3.0e) software based on Inorganic Crystal Structure Database (ICSD, Karlsruhe, Germany). The quantitative share of the identified crystalline phases was calculated using the Rietveld method.

**Figure 5:**
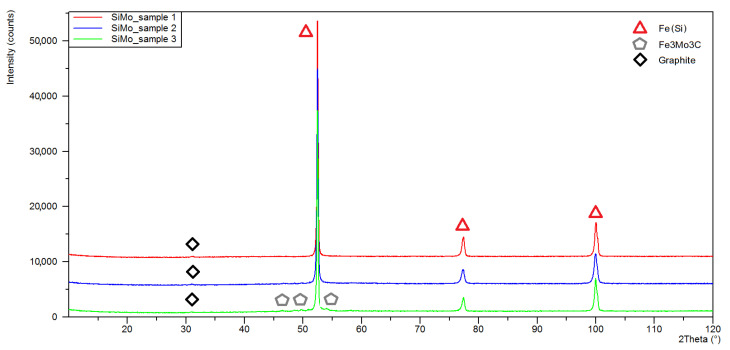
The X-ray diffraction patterns obtained for SiMo cast iron samples in the initial state; sample 1 (red line), sample 2 (blue line), sample 3 (green line).

Investigation of the phase composition of SiMo samples before the oxidation process (Figure 5), confirmed the share of the alpha iron (ferrite) phase. The recorded Feα diffraction peaks feature a shift towards higher angular values in relation to the pattern cards. It can be assumed that this is due to the presence of silicon atoms in the ferrite crystal lattice, which reduces the lattice parameter by 0.00185 Å for each Si wt%. In one article [33], it was shown that this coefficient curve is close to linear within the range of silicon share from 2.50 to 4.56 wt%. In all the diffractograms recorded for the initial state, the main graphite line (200) was also identified. Analysis of the diffractogram obtained for sample 3 (Figure 5, green line), containing 2% Mo, showed the presence of Fe_3_Mo_3_C phase. A higher Mo addition in cast iron promotes the formation of Fe-Mo-C-type carbide phases [34].

## 3. Results

### 3.1. XRD Analysis

**Figure 6:**
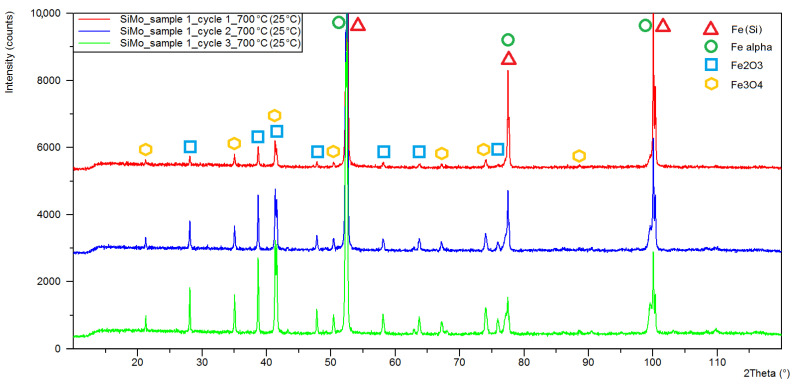
XRD patterns obtained for sample 1 of SiMo cast iron after one (red line), two (blue line) and three (green line) heating cycles at 700 °C.

**Figure 7:**
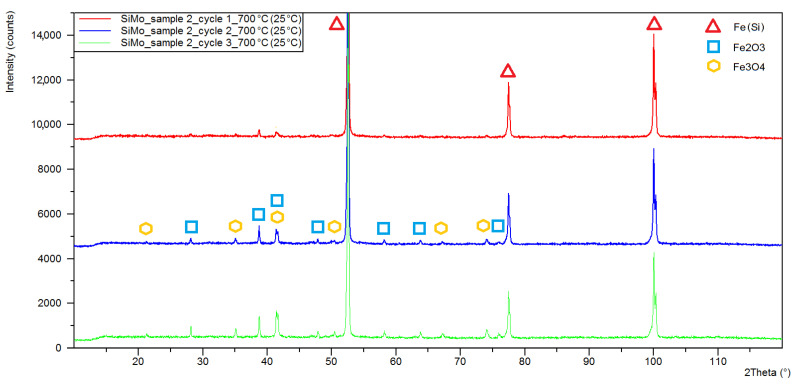
XRD patterns obtained for sample 2 of SiMo cast iron after one (red line), two (blue line) and three (green line) heating cycles at 700 °C.

**Figure 8:**
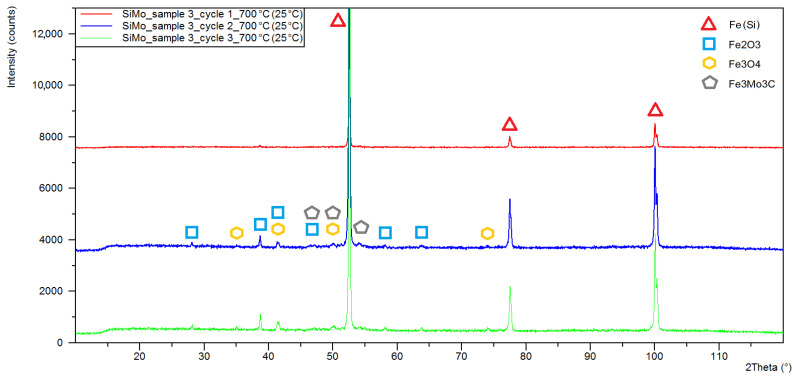
XRD patterns obtained for sample 3 of SiMo cast iron after one (red line), two (blue line) and three (green line) heating cycles at 700 °C.

Figure 6, Figure 7 and Figure 8 show the X-ray diffraction patterns of the tested samples. As a result of the high-temperature oxidation of SiMo cast iron, the layers of iron oxides were formed, Fe_2_O_3_—Hematite, crystallizing in the hexagonal crystal system (ICSD: 98-002-2505) and Fe_3_O_4_—Magnetite, crystallizing in a face-centred cubic (98-008-5806). Peaks from the oxide phases, magnetite and hematite, were identified for all diffraction patterns except for sample 3 after the first oxidation cycle, where no Fe_3_O_4_ peaks were observed. For all diffractograms, iron alpha peaks were identified with a slight shift towards higher angular values. By analysing the diffractogram from sample 1 (Figure 6), double peaks were observed in angular positions characteristic for iron alpha, showing a shift of angular values of around 0.1° and a difference in the lattice parameter of about 0.001 nm.

The results of the quantitative phase analysis of the tested samples are presented in Table 2. The highest quantitative share and the increase in oxide phases were displayed in sample 1, in which, after the first cycle, the hematite content was estimated at 12.4% while the magnetite level was 7.9%. After three cycles, the share of hematite and magnetite in sample 1 was 46.6 and 17.8%, respectively. The lowest share of oxide phases was characteristic for sample 3, in which the maximum determined contents of hematite and magnetite were at the levels of 9.8 and 2.9%.

### 3.2. SEM Analysis

**Figure 9:**
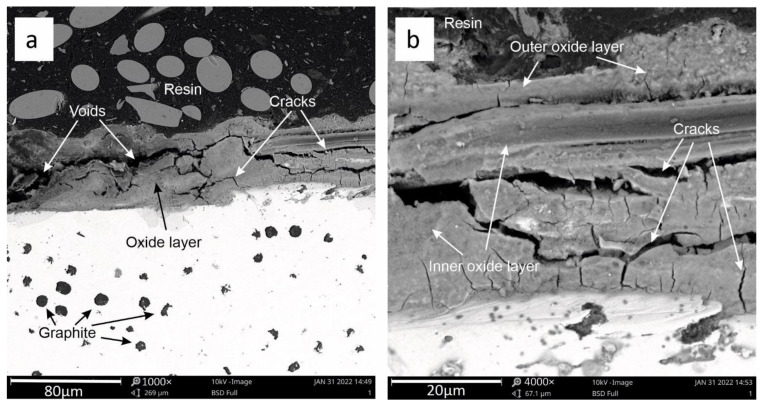
View of the oxide layer after three heating cycles (**a**) for sample 1. Detailed description of the oxide layer (**b**).

**Figure 10:**
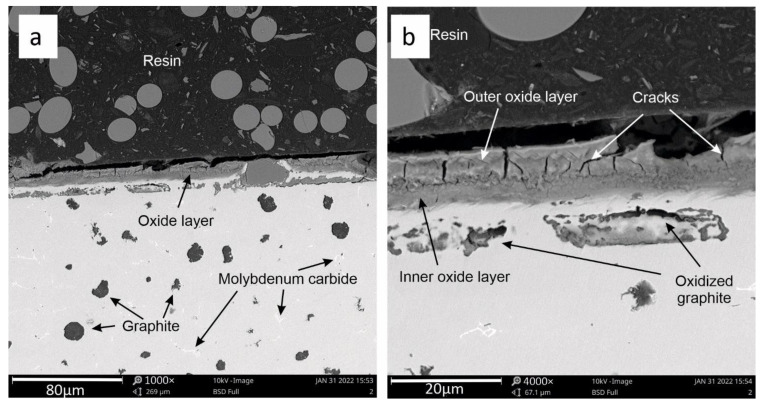
View of the oxide layer after three heating cycles (**a**) for sample 2. Detailed description of the oxide layer (**b**).

**Figure 11:**
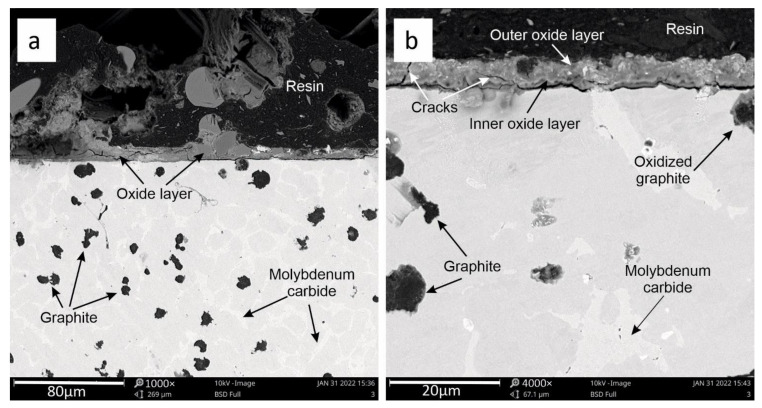
View of the oxide layer after three heating cycles (**a**) for sample 3. Detailed description of the oxide layer (**b**).

Figure 9, Figure 10 and Figure 11 show the visual analysis of the oxide coating quality. Figure 9a shows clearly visible cracks in the oxide coating. Corrosion products are distributed loosely and unevenly. This layer can be divided into two main parts: inner oxide layer and outer oxide layer—Figure 9b. The boundary between these layers is clear. In addition, the inner layer features multiple transverse cracks. In the oxide layer, under analysis, numerous voids are visible—marked in Figure 9a.

Figure 10a,b shows the oxide coating for sample 2. Its structure is different compared to the coating obtained in sample 1. A clearer division into the outer and inner layers is visible here. The internal (passive) layer described in [21] is a continuous coating in nature, showing a good contact with the sample material. The outer layer is looser and features visible transverse cracks. The connection of the inner layer with the outer layer is continuous. No defects, cracks, etc., were observed there. For sample 2, there are no voids in the oxide layer, as was the case for sample 1 (Figure 9a). In addition, oxidized graphite precipitates were observed in the near-surface layer of the metallic matrix.

Figure 11a,b shows the oxide layer for sample 3 (with the highest Mo content, of around 2%). The coherence of the oxide layer on the boundary between the outer layer and the inner layer is clearly visible here. There are few cracks in the oxide layer here.

The division into outer and inner oxide zones for sample 3 is not clear. As in the case of sample 2, oxidized graphite precipitates were observed here. They are marked in Figure 11b.

### 3.3. Oxide Layer Thickness Analysis

**Figure 12:**
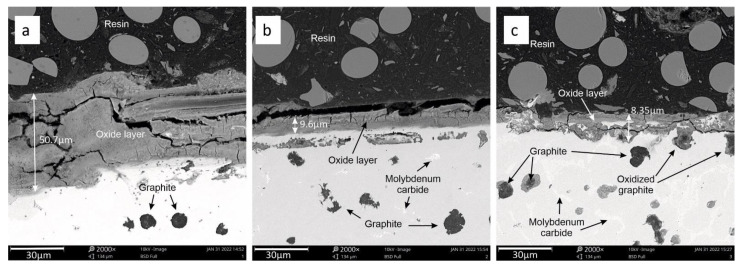
Thickness of the oxide layer for the samples under analysis. Sample 1 (**a**), sample 2 (**b**), sample 3 (**c**).

The results of metallographic analysis using scanning microscopy are presented below. An inbuilt tool was used to measure the geometry of the selected microstructure components. Figure 12a–c shows an example of measurement results of the oxide layer thickness for each of the samples under analysis.

The analysis of results presented in Figure 12a–c clearly shows that the thickness of the oxide layer for sample No. 1 is the highest, at around 50.7 µm (Figure 12a). The thickness of the oxide layer decreases with the addition of molybdenum (for sample 2) to a value of about 9.6 µm (Figure 12b). The lowest thickness of the oxide layer is found in sample 3. Its thickness is 8.37 µm (Figure 12c).

**Figure 13:**
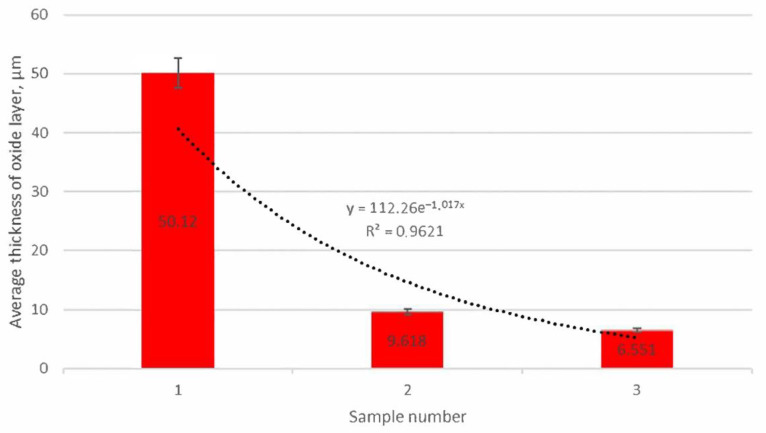
Average thickness of the oxide layer for the samples under analysis.

For each case under discussion, the thickness of the oxide layer was measured. On the basis of the obtained results, a graph was created (Figure 13) to show the average thickness of the oxide layer depending on the sample number.

It is clearly visible that there is a significant difference between the average thickness of the oxide layer for the samples under analysis. No molybdenum added (sample 1) caused a significant increase in the thickness of the oxide layer.

## 4. Discussion

**Figure 14:**
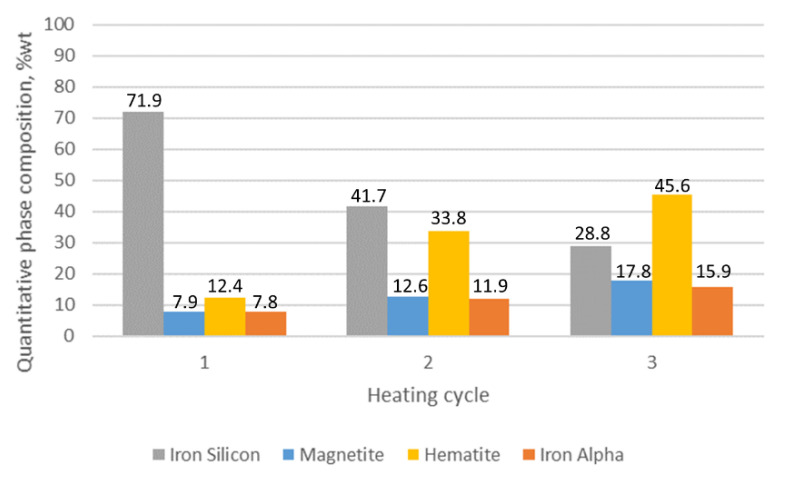
The quantitative share of outer oxide layer phase components for sample 1 as a function of heating cycles.

The conducted analysis allowed us to determine the growth dynamics of the outer oxide layer components for the alloys under analysis. For alloy without molybdenum addition (sample 1, Figure 14), we can clearly see that the share of the oxide layer components changes dynamically, mainly at the expense of silicon ferrite. Its share decreased by 50% in relation to other components. In the case under discussion, we are also dealing with a dynamic increase in the share of hematite reaching the level of 45.6%. The share of magnetite also increases 17.8%.

Visual analysis of the oxide-layer quality also revealed multiple cracks, voids resulting from the dynamic growth of this layer and poor resistance to temperature changes. The average oxide layer thickness in this case was over 50 µm.

**Figure 15:**
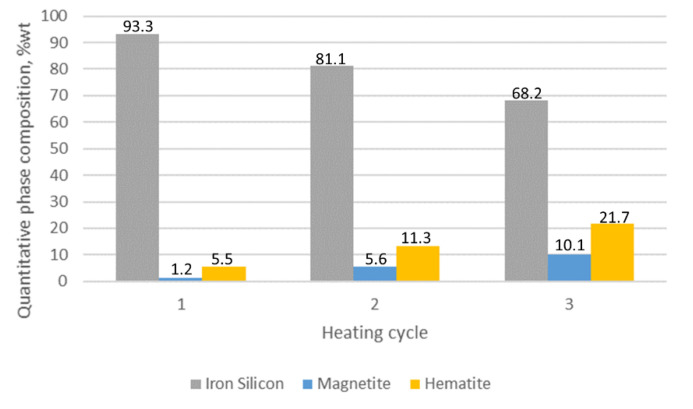
The quantitative share of outer oxide layer phase components for sample 2 as a function of heating cycles.

For the alloy with the addition of 1% Mo, the phases constituting the outer oxide layer structure include: silicon ferrite, magnetite and hematite. In the case under discussion, the decrease in the share of silicon ferrite is not as dynamic as it was in the case of sample 1. The decrease for the three heating and cooling cycles was 25.1% (Figure 15). The increase in the amount of the remaining components for the alloy with 1% Mo was also less intense compared to sample 1. The increase in the share of magnetite was 10.1%, while the share of hematite was 21.7%. This dependence, taking into account the specificity of the diffraction test, can be explained by the lower thickness of the oxide layers in relation to the X-ray penetration depth.

For the sample with the addition of 1% Mo, the average thickness of the oxide layer was 9.6 µm, which corresponds to a significant decrease in its thickness in relation to the sample 1 by about 80%. The above-described effect can be explained by the stabilizing effect of molybdenum on corrosive processes [6,7] and by reducing the alloy sensitivity to thermal shocks [4].

**Figure 16:**
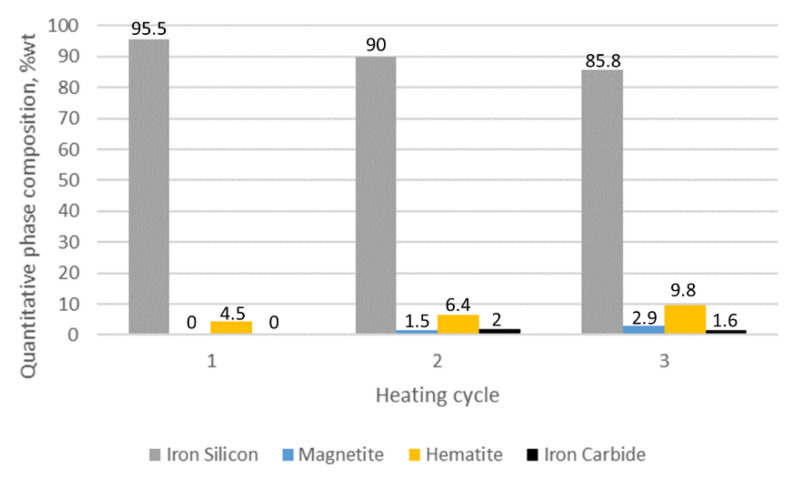
The quantitative share of outer oxide layer phase components for sample 3 as a function of heating cycles.

For the alloy containing 2% Mo, we did not observe such dynamic changes in the share of the surface layer phase components (Figure 16). The decrease in the share of silicon ferrite was 9.7%. We also observed a slight increase in magnetite, up to 2.9%, and hematite up to 9.8%. In the case under analysis, a new component in the form of Fe_3_Mo_3_C carbide was identified, and its share fluctuated around 2%.

The average thickness of the oxide layer for cast iron with 2% Mo addition is 6.55 µm. It is clearly visible that increasing the Mo addition above 1% does not result in a significant decrease in the corrosive layer thickness.

Increasing the Mo content in the alloy causes a decrease in the share of magnetite and hematite in the oxide surface layer. For the alloy without the addition of Mo, the magnetite share is 17.8%, for the alloy with the addition of 1% Mo it is 10.1%, and for the alloy with 2% Mo it is 2.9% magnetite. The same is true for hematite, whose share for the sample without Mo is 45.6%, for sample 2 (1% Mo) is 21.7%, while for sample 3 (2% Mo) is 9.8%. For cast iron with the addition of 2% Mo, Fe_3_Mo_3_C carbides were identified in the surface layer. However, the quantitative data obtained for the Fe_3_Mo_3_C phase may contain some increased uncertainty of calculations due to the low peak intensity.

In one study [35], C. K. Clayton and Y. C. Lu showed that the positive effect of molybdenum on corrosion resistance is associated with the formation of a permanent amorphous passive layer. According to Newman [36], molybdenum accumulates in active places, at the edges and corners of the lattice, while dissolving the alloy in the active state reduces the corrosion rate. Olefjord [37] suggests that hexavalent molybdenum is incorporated into the passive layer, eliminating local defects in it.

It should be noted that the increase in the corrosion resistance of cast iron is achieved by adding into cast iron elements with high electrochemical potential (Ni, Cu, Mo) or elements that make up the passive layer (Cr, Al, Si), which is consistent with the description contained in articles [4,5,6,7,8,9,10,11] To obtain the required corrosion resistance, the solid solution must contain a certain share of the alloying element. Therefore, factors that reduce the solubility of alloying elements in a solid solution by binding them into carbides, and also by increasing the degree of microsegregation of a given element, require neutralisation by increasing the overall share of the alloying element.

## 5. Conclusions

The conducted research allows us to draw the following conclusions:

- Adding molybdenum to silicon cast iron stabilises the corrosion process and reduces the thickness of the oxide layer.
- For the addition of 1% Mo, the decrease in the thickness of the oxide layer is 80% compared to the alloy without molybdenum.
- Increasing the Mo concentration from 1% to 2% causes a decrease in the oxide layer thickness by about 31% (compared to the alloy with 1% Mo addition).
- The reduction in the oxide layer thickness for the alloy with 2% Mo compared to the alloy without molybdenum addition is about 87%.
- For the alloy without molybdenum addition (sample 1), it is clearly visible that the oxide layer is formed with the decrease in silicon ferrite, the loss of which is 50%.
- For the sample with the addition of 1% Mo, the decrease in the share of silicon ferrite in the top oxide layer is 25.1%, while for the sample with the addition of 2% Mo, the decrease is only 9.7%.
- Increasing the Mo content in the alloy causes a decrease in the share of magnetite and hematite in the oxide surface layer.
- The 1% Mo concentration for cast iron provides an optimum-quality oxide layer.

## Figures and Tables

**Table 1:** Chemical composition of spheroidal cast iron.

Sample Number		Chemical Composition, % of Weight,						
	^1^ C	Si	Mo	P	^1^ S	Mg	^2^ C_eut_	Fe (Balance)
1	3.01	5.09	0.01	0.023	0.007	0.042	4.595	91.818
2	3.06	5.02	1.03	0.022	0.009	0.039	4.623	90.820
3	3.02	5.04	2.07	0.018	0.006	0.047	4.588	89.799

^1^ Carbon and sulphur analysis by CS 125 Leco, ^2^ eutectic carbon equivalent calculated for C, P, and Si.

**Table 2:** Results of quantitative phase analysis.

Sample Number Heating Cycle	Quantitative Phase Composition (%wt)				
	Iron (Si)	Iron Alpha	Magnetite	Hematite	Fe_3_Mo_3_C
1/C1 ^1^	71.9	7.8	7.9	12.4	-
1/C2 ^2^	41.7	11.9	12.6	33.8	-
1/C3 ^3^	28.8	15.9	17.8	45.6	-
2/C1 ^1^	93.3	-	1.2	5.5	-
2/C2 ^2^	81.1	-	5.6	13.3	-
2/C3 ^3^	68.2	-	10.1	21.7	-
3/C1 ^1^	95.5	-	-	4.5	-
3/C2 ^2^	90	-	1.5	6.4	2
3/C3 ^3^	85.8	-	2.9	9.8	1.6

^1^ C1—heating cycle No. 1, ^2^ C2—heating cycle No. 2, ^3^ C3—heating cycle No. 3.

## Data Availability

Data sharing is not applicable to this article.
